# The Rise and Fall of Plankton: Long-Term Changes in the Vertical Distribution of Algae and Grazers in Lake Baikal, Siberia

**DOI:** 10.1371/journal.pone.0088920

**Published:** 2014-02-25

**Authors:** Stephanie E. Hampton, Derek K. Gray, Lyubov R. Izmest'eva, Marianne V. Moore, Tedy Ozersky

**Affiliations:** 1 National Center for Ecological Analysis and Synthesis, University of California Santa Barbara, Santa Barbara, California, United States of America; 2 Scientific Research Institute of Biology, Irkutsk State University, Irkutsk, Russia; 3 Department of Biological Sciences, Wellesley College, Wellesley, Massachusetts, United States of America; Stazione Zoologica, Italy

## Abstract

Both surface water temperatures and the intensity of thermal stratification have increased recently in large lakes throughout the world. Such physical changes can be accompanied by shifts in plankton community structure, including changes in relative abundances and depth distributions. Here we analyzed 45 years of data from Lake Baikal, the world's oldest, deepest, and most voluminous lake, to assess long-term trends in the depth distribution of pelagic phytoplankton and zooplankton. Surface water temperatures in Lake Baikal increased steadily between 1955 and 2000, resulting in a stronger thermal gradient within the top 50 m of the water column. In conjunction with these physical changes our analyses reveal significant shifts in the daytime depth distribution of important phytoplankton and zooplankton groups. The relatively heavy diatoms, which often rely on mixing to remain suspended in the photic zone, shifted downward in the water column by 1.90 m y^-1^, while the depths of other phytoplankton groups did not change significantly. Over the same time span the density-weighted average depth of most major zooplankton groups, including cladocerans, rotifers, and immature copepods, exhibited rapid shifts toward shallower positions (0.57–0.75 m y^−1^). As a result of these depth changes the vertical overlap between herbivorous copepods (*Epischura baikalensis*) and their algal food appears to have increased through time while that for cladocerans decreased. We hypothesize that warming surface waters and reduced mixing caused these ecological changes. Future studies should examine how changes in the vertical distribution of plankton might impact energy flow in this lake and others.

## Introduction

Climate change is significantly impacting freshwater ecosystems worldwide. Recent studies indicate that many lakes are experiencing physical changes that include warmer surface water temperatures, altered water levels and wind patterns, longer ice-free periods, altered thermal stratification, and changes in water transparency [1]–[3]. Ecologists are beginning to understand the direct and indirect effects of these physical changes on biological communities [4]. Some of the documented responses of plankton to climate change include changes in abundance, phenology, body size, community structure, life history parameters, and vertical migration patterns (reviewed in [4]).

Altered thermal stratification is one of the most consequential indirect pathways through which climate affects plankton. Stratification not only provides vertical thermal structure, but it also alters the distribution of nutrients and plankton [5]–[7]. During periods of summer stratification lakes are often separated into a warm, shallow, well-lit epilimnion and a deep, cool hypolimnion that receives less solar energy. As time passes after the onset of stratification, nutrient availability can become reduced in the upper stratum due to the lack of vertical mixing that brings nutrients up from the hypolimnion [8]–[10]. Heavier plankton and those without buoyancy or mobility mechanisms may sink away from the upper waters where light is most readily available [5], [11].

The effects of climate change on summer stratification can be highly system-specific, complicating ecological predictions [12]. However, two decades of modeling studies and empirical observations of deep northern temperate lakes indicate that climate change is altering stratification [13]. In general, the length of the stratification period and thermal stability has increased through time [12], [13]. These changes have been linked to observed shifts in plankton communities [13]. For example, in Lake Tahoe surface waters the algal community has shifted toward small, slow-sinking taxa as turbulent mixing has decreased over the past 23 years, with a downward shift of relatively heavy diatoms [11].

While previous studies have explored the effects of increased thermal stability on the depth distribution of phytoplankton species, few have explored how zooplankton might respond to changes in stratification (reviewed by [4]). The vertical position of zooplankton in the water column frequently exhibits a diurnal pattern whereby individuals are found in deeper waters during the day but migrate closer to the surface at night [14], [15]. This vertical migration is thought to be a behavioral adaptation that balances the risk of predation from visually orienting predators with the potential benefits of inhabiting the epilimnion, such as access to food and the metabolic benefits of warmer ambient temperature.

There is now a large body of literature examining the factors driving daily variation in zooplankton depths (i.e. the factors responsible for vertical migration), but less attention has been given to vertical positioning across seasons or years. The few studies that have examined seasonal differences in zooplankton vertical distribution suggest that stratification plays an important role. Thackeray et al. [16], [17] found that the onset of stratification and the vertical position of the thermocline were both related to zooplankton depth distributions. Several other seasonal studies reported that zooplankton tend to shift to shallower positions when summer stratification sets in [18]–[20]. In a more direct test of the impact of thermal structure, Marcogliese and Esch [21] demonstrated that artificially deepening the epilimnion caused simultaneous changes in the depth distribution of zooplankton. Taken together, the previous work indicates that alterations in stratification due to climate change may have strong effects on the depth distribution of zooplankton.

Subarctic Lake Baikal may be especially sensitive to changes in mixing patterns. The lake is covered with ice for almost half the year, from January to May, with stratification occurring weakly for about 6 to 8 weeks in August and September [22], [23] and also under the ice in winter [24]. Density gradients are relatively low at Lake Baikal's low water temperatures, and summer stratification is readily broken down by upwellings, storms, and wind events [25], [26]. Thus the dominant plankton are well adapted to mixed, dynamic environmental conditions. However, like many other lakes worldwide, Lake Baikal has experienced dramatic warming. The ice-covered period is shorter and ice thickness has decreased [27]. Warming has been strongest in the summertime, and in the upper stratum [28], [29]. Warming is not yet manifest in deeper waters (>50m), implying that summer stratification should be stronger, and thus may have the potential to last longer [28]. These ongoing changes in surface temperatures and thermal stratification are expected to lead to a shift in pelagic phytoplankton communities away from one dominated by the coldwater diatoms *Aulacoseira baicalensis* and *Cyclotella minuta* to one dominated by green and cyanobacteria picoplankton [30], [31].

In this study we use 45 yr of data from Lake Baikal to examine how the depth distribution of major zooplankton and phytoplankton groups has changed through time. In addition, we explore the implications that changes in depth distributions may have for interactions between phytoplankton and their zooplankton grazers. Our results provide further evidence that significant long-term changes are occurring in Lake Baikal's plankton community and that these changes are likely driven by climate.

## Methods

Data used in the study are part of a historic Russian data set, registered with the Russian government (No. 2005620028). No endangered, protected, or vertebrate species were targeted in those sampling efforts. No contemporary data were collected for this study.

**Figure 1:**
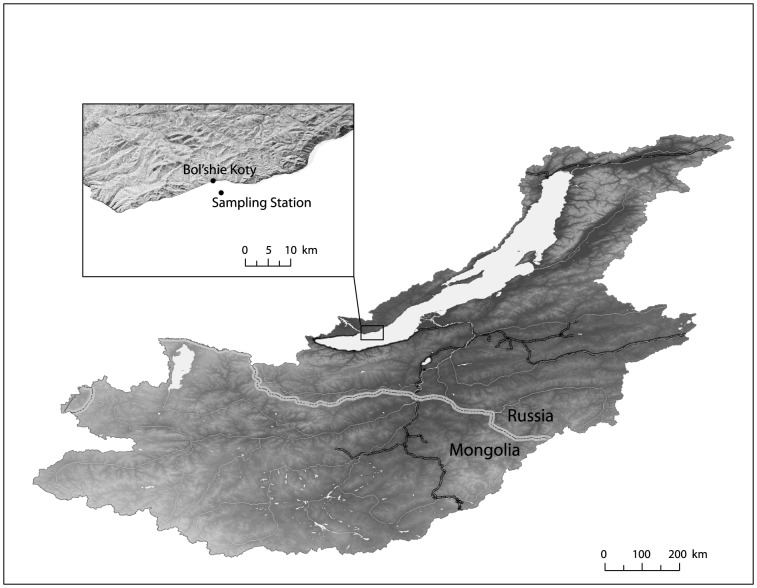
Map of Lake Baikal and the long-term Irkutsk State University sampling station.

Since 1945 researchers from Irkutsk State University (ISU) have collected daytime plankton, temperature and Secchi depth data at least monthly, usually every 7–10 days, in depth profiles from the surface to at least 250 m at a single main station approximately 2.7 km offshore from Bol'shie Koty in the Southern Basin (Fig. 1). This station is not influenced by discharge from the Baikalsk pulp mill, more than 80 km to the south [22], [32]. While limitations are presented by analyzing data from a single station, trends in plankton abundance at this station are similar to those reported for a second location in the Southern basin [33]. Sampling did not occur during crepuscular hours, as diel vertical migrations are well known for many Baikal plankton. We have focused our analyses on the summer months in which stratification most frequently occurs – July, August, and September.

Temperature was measured with a mercury thermometer in water collected at discrete depths by a 10 L Van Dorn bottle; those measurements used here were from depths of 0, 10, 50, 100, and 200 m. Phytoplankton samples obtained at these same depths with the Van Dorn bottle were preserved before settling in Utermöhl chambers. A change in phytoplankton preservation, from the use of formalin to a Lugol's solution in 1973, complicated our analysis, so unless otherwise stated our analyses include only phytoplankton data from 1975 forward, allowing a conservative buffer for the adjustment to the new protocol. There are no obvious effects of the preservation change on diatom data, so we have examined diatom records beginning in 1964 when sampling became consistent across depths and over time.

Single zooplankton samples were collected with a closing plankton net (37.5 cm diameter, 100 µm mesh) from depth layers of 0–10, 10–25, 25–50, 50–100, 100–150, 150–250, and 250–500 m. Samples from the 25–50 and 250–500 m depth layers were excluded from our analyses because sampling frequency was least consistent at these depth layers across the time series. The 100 µm mesh may not sample smaller individuals such as some rotifer species and age classes, so these results should be interpreted cautiously. Zooplankton samples were fixed in formalin throughout the duration of the long-term monitoring program with greatest consistency of temporal and spatial sampling occurring from 1955 forward, the years included in these analyses. Both phytoplankton and zooplankton were identified and counted at the species level, and copepods were enumerated by age class, following a subsampling protocol that was consistent since the inception of ISU's research program [22] in which subsamples are examined until at least 100 individuals of each species or age group are observed. The zooplankton community in the open water is dominated by the herbivorous copepod *Epischura baikalensis*, comprising approximately 90% of zooplankton biomass [34].

### Temperature and light environment

Vertical resolution of the temperature data did not allow determination of the thermocline depth or a quantitative evaluation of stratification. As an alternative metric for the conditions under which stratification likely occurred, we calculated relative thermal resistance to mixing (RTRM; [35]) 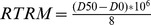 based on the density (*D*) of water at 0 m and 50 m temperatures (*T*), where 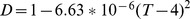.

Secchi depth (m) was used to estimate the depth of the photic zone (PZ), the depth to which 0.1% surface light penetrates, using the classic relationship described in Cole [36] that has been used in previous Baikal research [22], [37]. The light extinction coefficient is calculated as 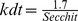 and the depth of the photic zone is then calculated as 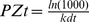.

To describe the average light environment experienced by each phytoplankton group, we calculated a density-weighted exposure to light (DWL) for each phytoplankton taxon *i* with abundance *n* at time *t* using the percentage of light (*l*) at each depth: 
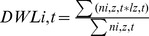
.

### Plankton depth and distribution

The average depth of each taxonomic group was calculated as a density-weighted average depth [38] 
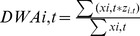
 where *x* is the abundance of each taxon *i* at depth *z* on a given date *t*. For zooplankton, the depth at the midpoint of the vertical tow was used as *z_i_*. Dates on which samples were not collected at all five depth intervals were excluded from analyses.

We examined the trajectory of DWA through time for five zooplankton taxonomic/lifestage groups and seven phytoplankton groups (Table 1). A general linear model with density-weighted depth as the response variable and year and taxa as fixed factors was implemented in R using the lm{stats} function. Zooplankton and phytoplankton were analyzed separately (i.e. two models were used). To test whether trends differed from zero we performed general linear hypothesis tests using the glht{multcomp} function that corrects p-values for multiple comparisons [39]. Durbin-Watson tests conducted on individual least squares model fits for each taxon/lifestage group suggested that there was significant temporal autocorrelation in the residuals for adult copepods, cyanobacteria, cryptophytes, and green algae. Generalized linear models that incorporated an autoregressive parameter were also implemented for these taxa, but the statistical conclusions did not differ from the standard linear model (results not presented).

**Table 1:** Annual change in density-weighted depth for phytoplankton and zooplankton taxa.

Group	Taxa	Trend (y-^1^)	p-value
Phytoplankton	Chrysophyte	−0.0951	1.000
	Cryptophyte	−0.6896	0.8074
	Cyanobacteria	1.3168	0.1191
	Diatom	1.8861	0.0056
	Dinoflagellate	−0.2705	0.9988
	Green	0.9126	0.5154
	Picoplankton	0.4657	0.9704
Zooplankton	Copepodites	−0.6830	0.0001
	Nauplii	−0.6269	0.0004
	Adult copepods	−0.0303	0.9999
	Rotifers	−0.7532	1.55*10^−5^
	Cladocerans	−0.5705	0.0092
Overlap with all phytoplankton	Copepodites	−1.8070	0.0198
	Nauplii	−1.2787	0.2062
	Adult copepods	−1.8137	0.0191
	Rotifers	−1.5472	0.0696
	Cladocerans	−1.7312	0.0291
	All zooplankton	−1.6012	0.0544

Trend was estimated from the general linear model and P-values were obtained from general linear hypothesis tests (see Methods). Negative or positive trend values for phytoplankton and zooplankton indicate that they are moving shallower or deeper, respectively.

To evaluate if and how the spatial overlap of zooplankton and phytoplankton changed, we calculated the difference between the DWA of zooplankton and that of phytoplankton through time. For this analysis the DWA for each of the five zooplankton taxon/life stage groups (Table 1) was compared with the overall DWA for all phytoplankton groups combined. The significance of the trends in the difference between phytoplankton and zooplankton DWA through time was explored using the methods described above for DWA – a general linear model combined with general linear hypothesis tests. Other modes of calculating overlap (e.g. [40]) yielded similar results (unpublished results) but were considered less appropriate because of the difference in sample collection of zooplankton (stratum sampled by closing net) and phytoplankton (discrete depths sampled by bottles).

### Trends in plankton abundance by depth

For plankton groups that exhibited significant changes in DWA we also analyzed trends in abundance by depth interval. To do this we calculated the annual mean abundance of each group in summer (July, August, September) at each depth. Trends were examined with a general linear model combined with general linear hypothesis tests, following methods described above for DWA.

## Results

**Figure 2:**
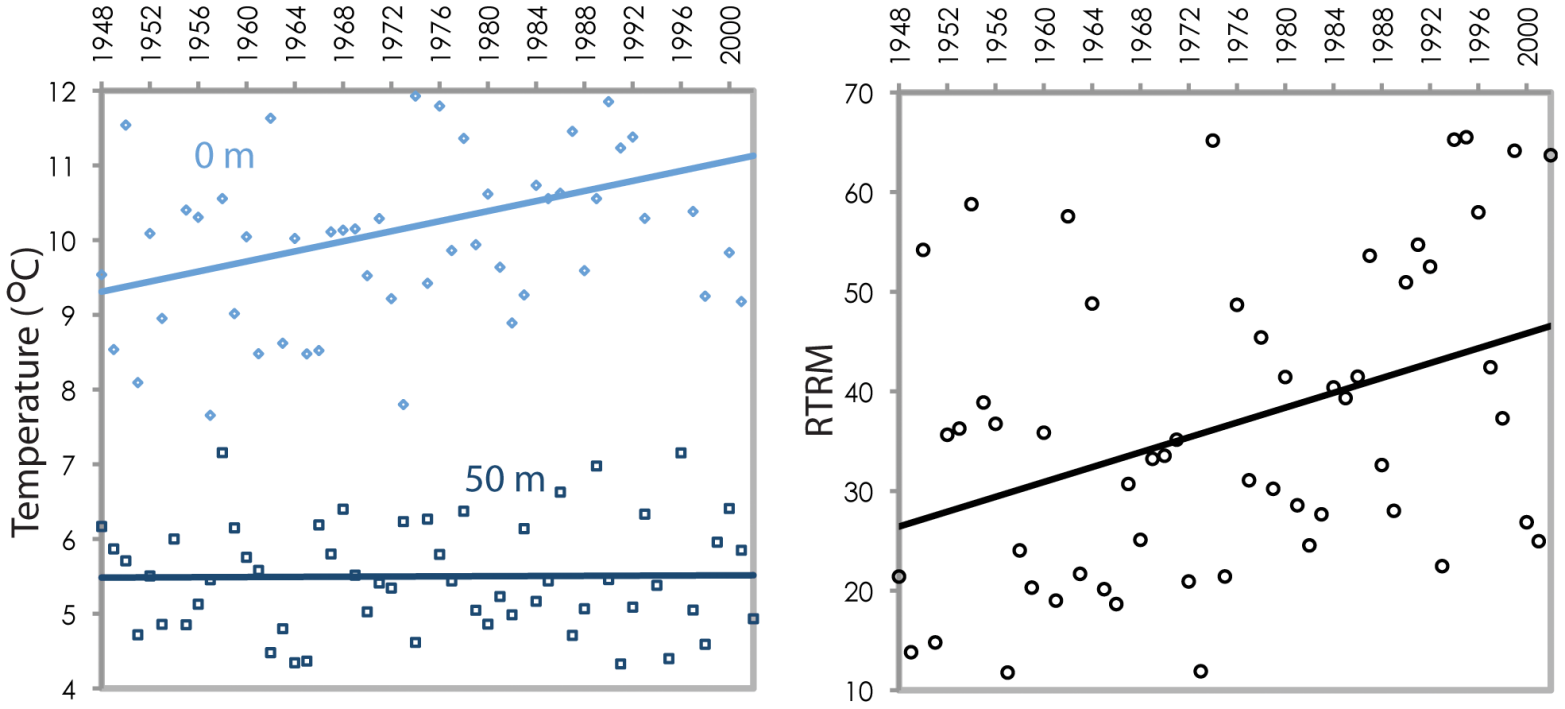
Relative thermal resistance to mixing (RTRM) estimated from average temperature (July, August, September) at 0 and 50 m from 1948 – 2002. Although vertical temperature profiles did not contain sufficient depth resolution for discerning thermocline depth or calculating a stratification index, temperature and density differences (calculated on each sampling date and averaged) implied increasing stratification across the time series, as represented by relative resistance to thermal mixing. RTRM is based on the difference in estimated water density at 0 m and 50 m temperatures, relative to the standard water density difference at 4° C and 5° C. The RTRM line was fitted with linear least-squares regression (R^2^ = 0.135, p = 0.003).

**Figure 3:**
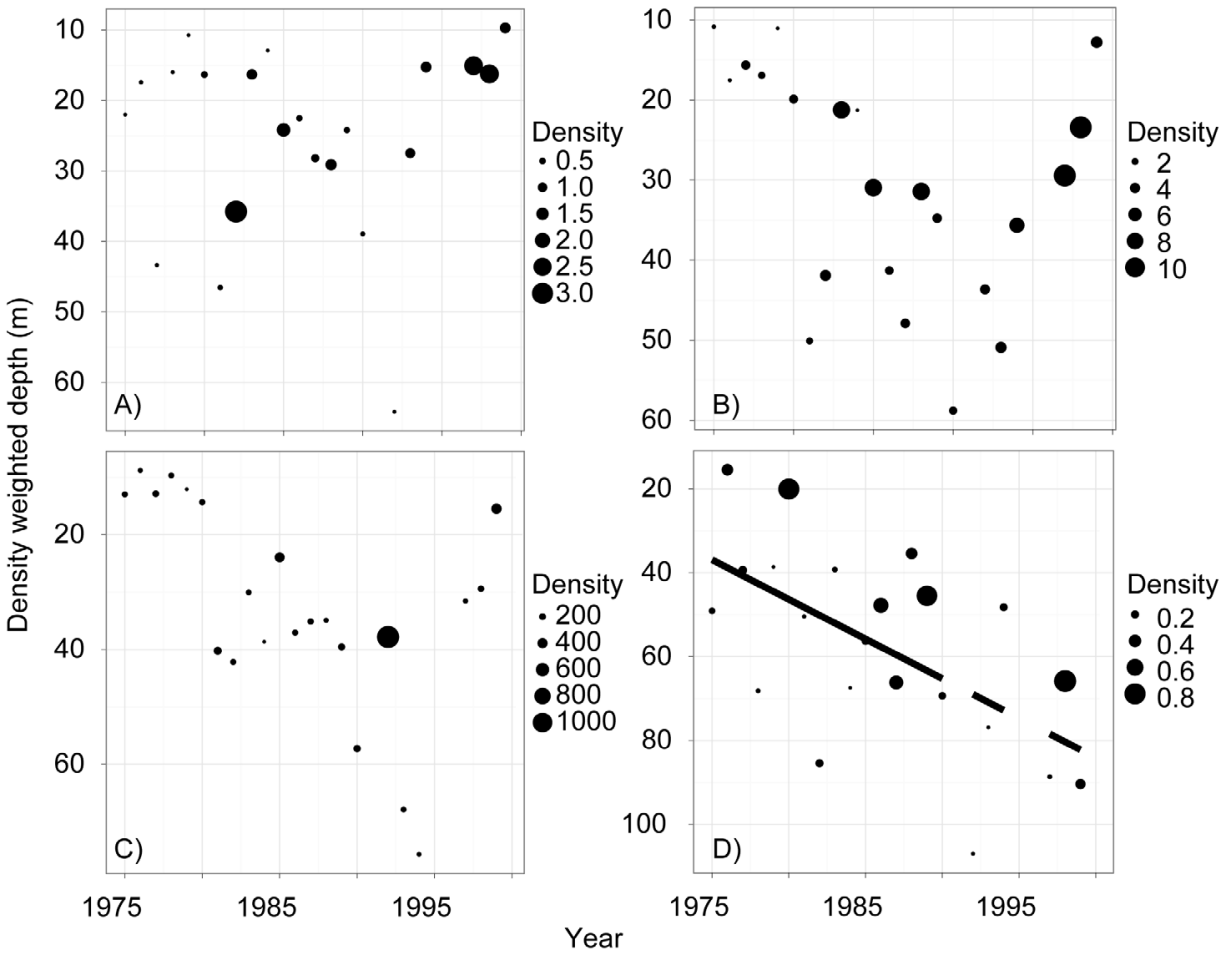
Abundance and density-weighted average summer depth of phytoplankton groups. Panels: A = Chrysophytes; B = Cryptophytes; C = Cyanobacteria; D = Diatoms. Cyanobacteria does not include picoplankton, which can be important contributors to Lake Baikal primary productivity in the summer, but are not routinely measured. Bubble size indicates average abundance (1000 cells l^−1^).

**Figure 4:**
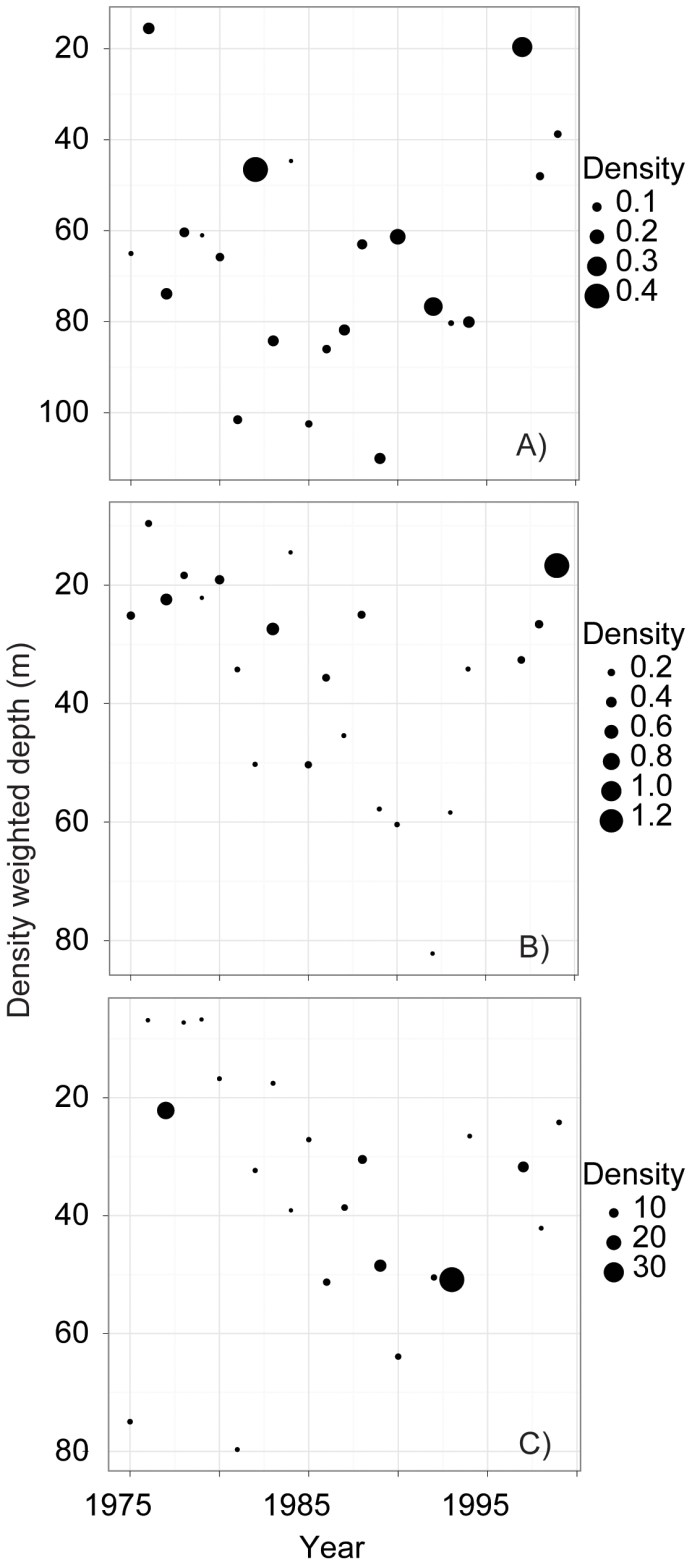
Abundance and density-weighted average summer depth of phytoplankton groups. Panels: A = Dinoflagellates; B = Green Algae; C = Unidentified Picoplankton (1000 cells l^−1^). Unidentified Picoplankton are unidentified cells at the limits of detection using standard methods in light microscopy, approximately 1–2 µm. Bubble size indicates average abundance (1000 cells l^−1^).

Temperature profiles suggested that summer stratification changed in Lake Baikal, with the temperature gradient between the surface and 50 m becoming stronger through time, resulting in significantly increasing relative thermal resistance to mixing (Fig. 2). The average summer surface temperature during this time period was 10.7° C (with maxima sometimes reaching 19° C), while the average summer temperature at 50 m was 5.5° C. Among the phytoplankton, cyanobacteria numerically dominated (Figs. 3 and 4); however, it is important to recognize that small picoplankton (<2 µm) were not included in the long-term data set, and they can be very abundant in Baikal during summer months [41], [42]. The abundance of small and mobile phytoplankton increased in the summer months over time, and this change was particularly notable for cryptomonads and chrysophytes (Figs. 3 and 4).

**Figure 5:**
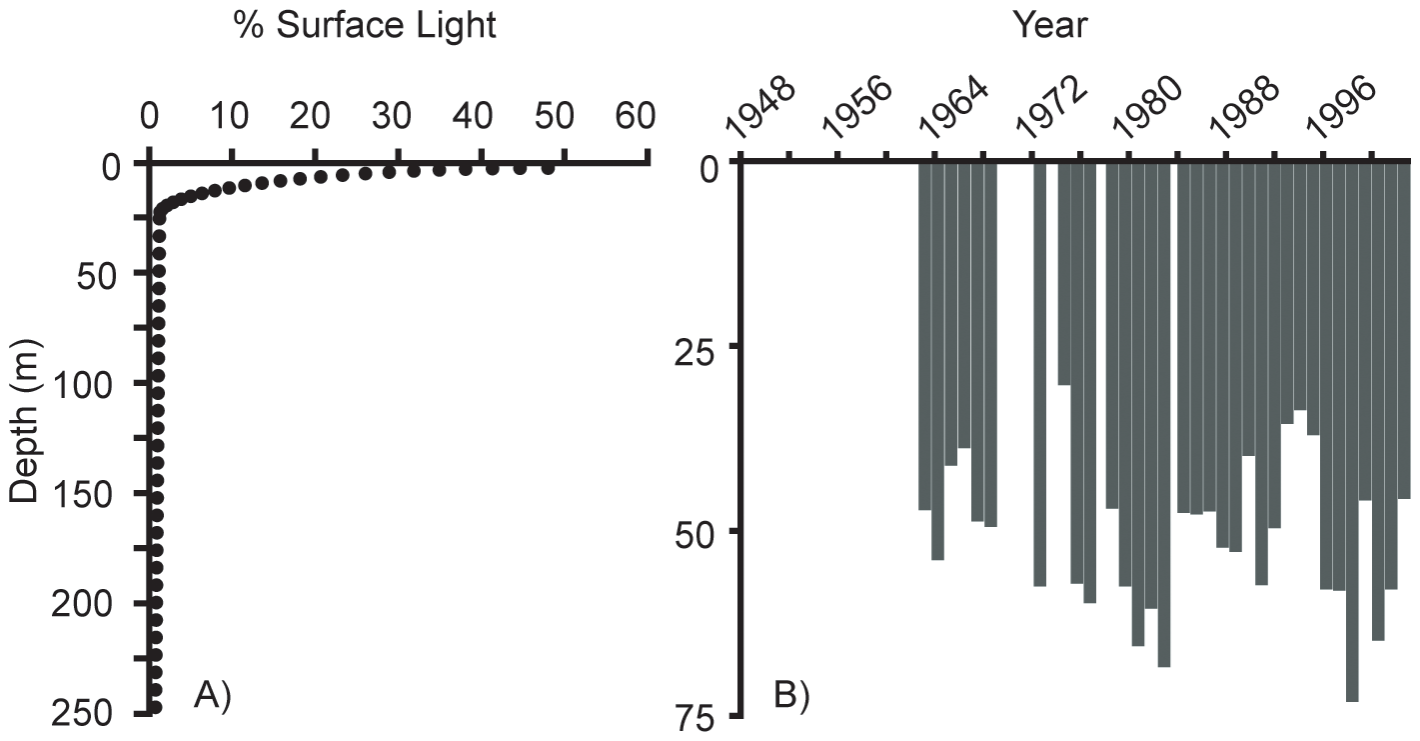
Average light extinction curve and photic zone in Lake Baikal. Light extinction with depth is shown in panel A, with the depth of the average photic zone (0.1% surface light) for each year in panel B.

**Figure 6:**
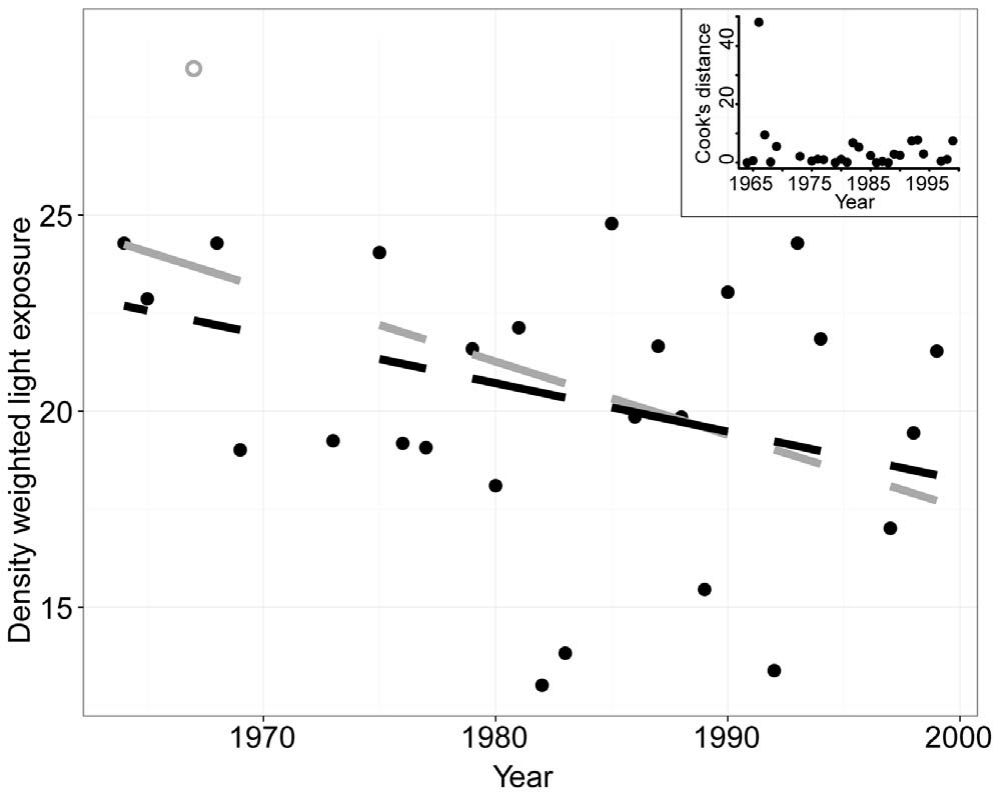
Change in density-weighted light exposure through time for diatoms between 1964 and 1999. Secchi data are available from 1964 forward, and diatom records are available beginning in 1951. A preservation change occurred in 1973, from formalin to Lugol's, and while a step change in diatom abundance is not apparent, results should be interpreted cautiously. The linear regression was fit with (grey line; P = 0.02) and without (black line; P = 0.09) the data point for 1966 (grey open circle). Cook's distance was also plotted for each point (inset), demonstrating the importance of the 1966 data point in influencing the results of the regression.

The average depth of diatoms increased across the time series while the depth of other phytoplankton groups did not change significantly (Table 1, Figs. 3 and 4). However, the depth changes for diatoms occurred at a shallow inflection point on the depth-light curve (Fig. 5), such that the net effect may not yet result in a significant decrease in light availability (Fig. 6) – i.e., diatoms were already in low light conditions on average, and at these depths light changes slowly.

**Figure 7:**
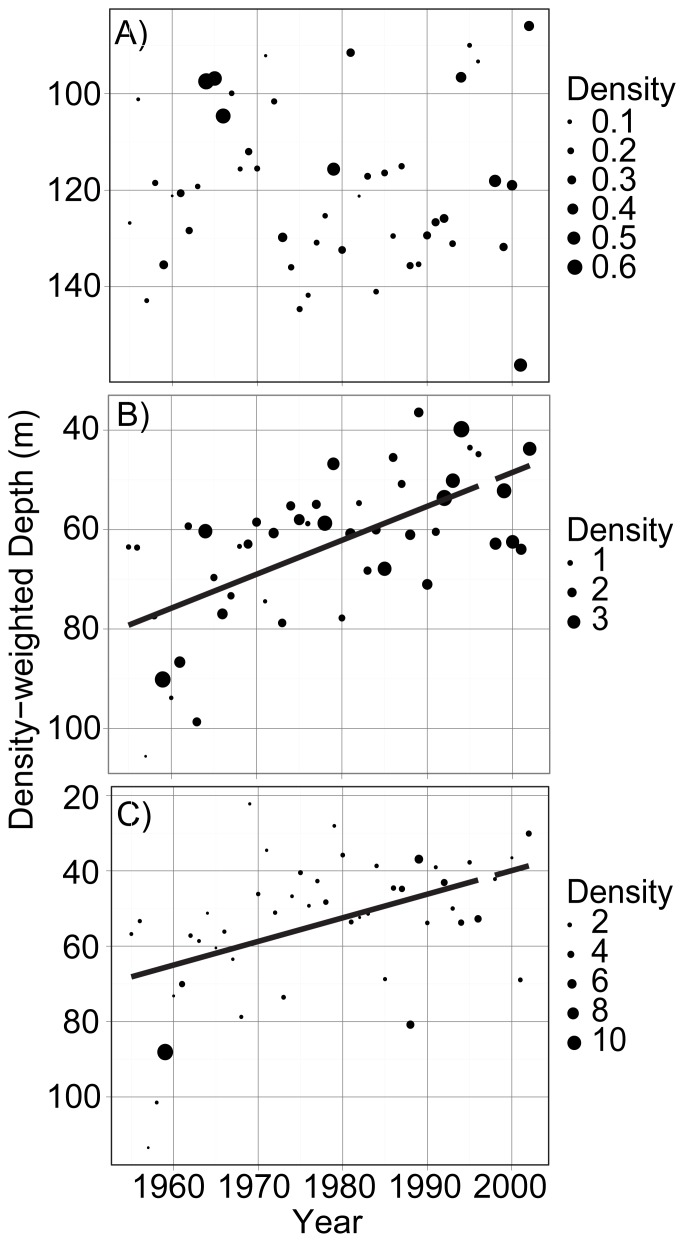
Abundance (individuals L^−1^) and density-weighted average depth of copepods in Lake Baikal through time. Panel A = Adult copepods; Panel B = Copepodites; Panel C = Nauplii.

**Figure 8:**
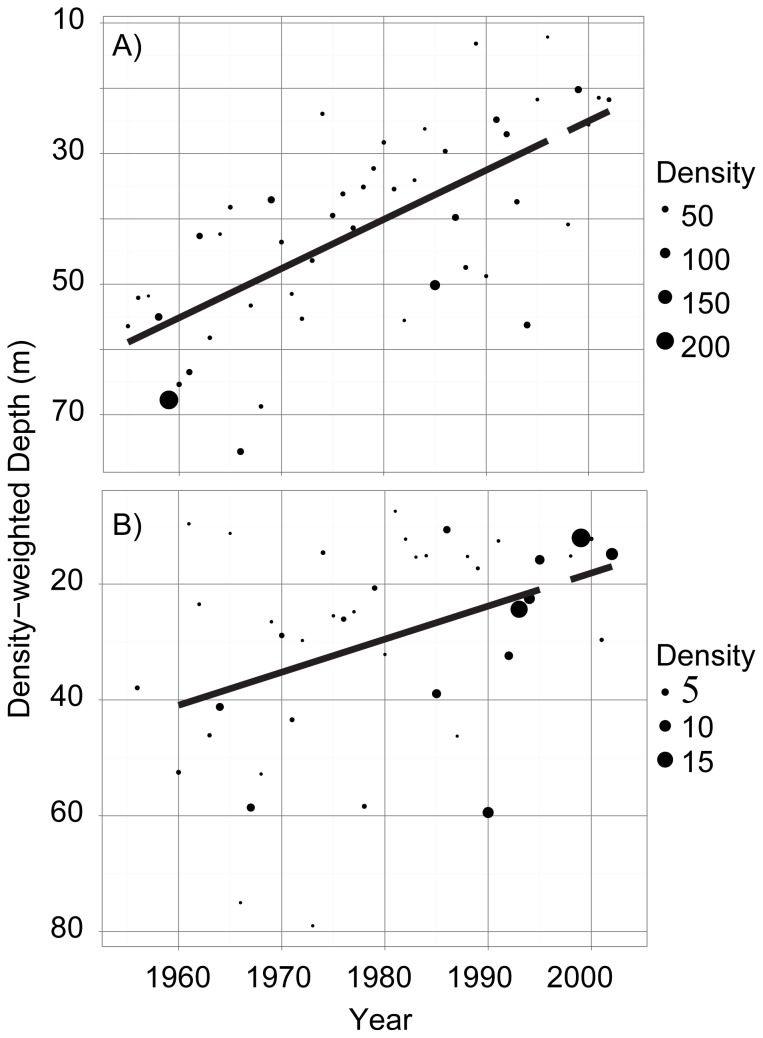
Abundance and density-weighted depth of rotifers (Panel A) and cladocerans (Panel B) in Lake Baikal.

Changes in zooplankton depth were dramatic; over time several important taxa moved from average depths below 60 m to average depths as shallow as 20 m (Table 1, Figs. 7 and 8). The dominant and endemic grazer *Epischura baikalensis* shows an interesting ontogenetic change in its depth distribution, with copepodites and nauplii moving into shallower water over time while adult *Epischura* remained primarily in deep water (Fig. 7). Rotifers and cladocerans also shifted significantly toward the surface (Fig. 8). At the same time that the distributions of these groups became shallower, the densities increased at shallow depths for copepodites, nauplii, rotifers (0–10 m and 10–25 m; P<0.05 for all), and cladocerans (0–10 m; P<0.05) and remained unchanged at deeper depth layers.

**Figure 9:**
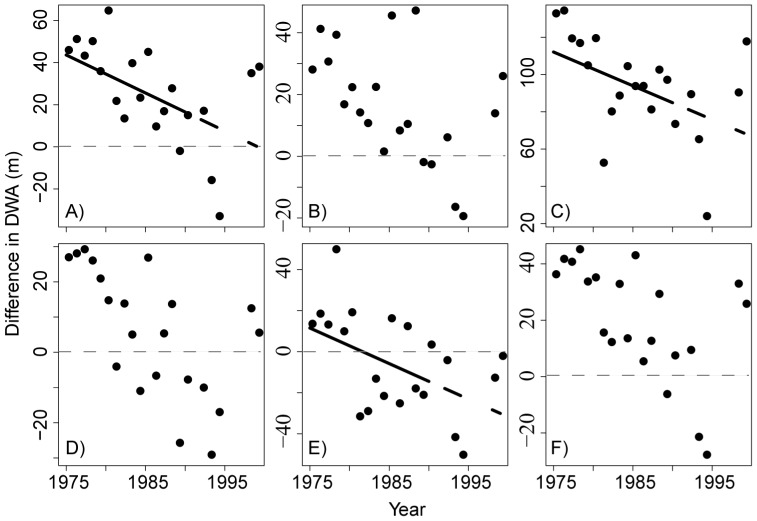
Difference in density-weighted average depth of zooplankton groups with all phytoplankton from 1974-1998. Zooplankton groups include copepodites (A), nauplii (B), adult copepods (C), rotifers (D), and cladocerans (E). Panel F shows all zooplankton combined versus all phytoplankton. A y-value of zero (dashed line) indicates that zooplankton and phytoplankton have the same density-weighted depth, while positive and negative values indicate that groups are deeper or shallower than the phytoplankton, respectively.

The shift of zooplankton towards the surface combined with phytoplankton groups either shifting deeper (diatoms) or not changing significantly with depth altered the spatial overlap of zooplankton grazers with the food resources analyzed here (Fig. 9), recognizing that picoplankton are probably not well represented in this data set. Spatial overlap appears to have increased for copepods, with distance between the DWA of the copepod groups and that of their food sources narrowing over time, while spatial overlap decreased for cladocerans through time (Fig. 9). Rotifers achieved greatest overlap with phytoplankton in the mid-1980 s and then continued to shift to shallower depths throughout the late 1980 s and 1990 s, leading to little absolute change in the amount of spatial overlap (Fig. 9) with algal resources.

## Discussion

Observed changes in phytoplankton distribution were consistent with predictions of stronger stratification as summer lake temperature warms. The average depth of the relatively heavy and non-motile diatoms became deeper over time (Fig. 3), while the depth of other phytoplankton taxa did not show strong linear change (Figs. 3 and 4). These changes are consistent with the hypothesis that stratification became stronger over time, disfavoring taxa such as diatoms that rely on mixing for suspension in the photic zone [43], [44]. Similar results were reported from Lake Tahoe, where large diatom species occupied deeper depths through time as stratification became stronger [11]. Size data specific to Lake Baikal diatoms are difficult to obtain, but measurements from other systems suggest that the most abundant diatoms in the lake fall into the “medium” (15–40 µm maximum linear dimension) or “large” size categories (>40 µm) described by Winder et al. [11]. In fact, four of the five most abundant species during the summer months have maximum lengths that exceed 15 µm, including *Synedra acus*, *Aulacoseira baicalensis*, *Nitzschia acicularis*, and *Cyclotella minuta* [45], [46]. While many of the Baikal diatom taxa are relatively large and heavy, some do have mechanisms for reducing sinking such as polysaccharide threads [47] and probably are variously susceptible to sinking under altered mixing regimes.

With the exception of adult copepods, the density-weighted average depths of all major zooplankton groups have become shallower through time (Figs. 7 and 8). These shifts in average depth distribution were remarkably rapid with copepod nauplii and copepodites, for example, shifting by 0.68 and 0.62 m per year, respectively. Given that abundances of copepodites, nauplii, rotifers, and cladocerans increased at shallow depths but remained unchanged at deeper depth intervals, changes in DWA seem unlikely to be driven by an exodus from deep waters. Instead, they may have been driven by a preference for shallower depths concurrent with an increase in the overall abundance of these zooplankton groups.

There are several possible proximate explanations for these long-term changes in zooplankton average depth distributions, each of which could have a multitude of ultimate causes. First, abundance may have increased within just the upper stratum, without behavioral shifts occurring. Second, changes in the daytime depth distribution could be influenced by changes in zooplankton vertical migration behavior. During the daylight hours most zooplankton species in Baikal shift to a deeper position in the water column to avoid visually orienting predators [48], [49]. If the occurrence or extent of vertical migration changed, then this could result in shallower daytime depth distributions. Alternatively, zooplankton may have shifted shallower while maintaining the same day:night variance around their mean depth. Unfortunately, we do not have night samples that would allow us to determine which scenario is more likely.

If zooplankton shifted their mean depth without altering the extent of their daily vertical migration behavior, then an obvious explanation might be that they are trying to maintain spatial overlap with phytoplankton food resources. However, this does not appear to be the case as our analyses indicate that zooplankton shifted shallower while phytoplankton did not. Another possibility is that they shifted to take advantage of picoplankton, a group that is not well represented in the sampling program due to their small size. In summer months, picoplankton represent between 10–50% of the primary production in the pelagic zone of Lake Baikal, and they are most abundant at higher temperatures and at depths less than 50 m [42]. In addition, past studies found that picoplankton are an important food source for some zooplankton species [50]. To our knowledge no long-term data on Baikal picoplankton exist, so we are unable to evaluate this hypothesis. Another alternative explanation is that zooplankton responded to rising surface temperatures. Our analyses suggest a significant change in the temperature gradient in the top 50 m of the water column through time, and previous studies reported that many zooplankton species shift to shallower positions during the warmest months of the year [18]–[20]. Analyses of the Lake Baikal zooplankton community conducted in the 1950 s and 1960 s indicated that most zooplankton in Baikal were already concentrated at depths less than 50 m during the summer months [22], so it is possible that rising surface temperatures provided a stronger cue for zooplankton to move upward.

If the explanation for zooplankton depth changes lies in modifications to vertical migration behavior, then some insights might be found in the rich literature on this subject. Many factors influence the occurrence and extent of zooplankton vertical migration including not only temperature gradients, but also light penetration, competition, predation, and the depth of the mixed layer [51]. In Lake Baikal there is no indication that summertime light penetration has changed through time [28]. Long-term changes in competition and predation regimes are possible, but we lack abundance data for important zooplankton predators including planktivorous fish (omul [*Coregonus autumnalis migratorius*], golomyanka [*Comephorus* spp.], and other pelagic sculpins [*Cottocomephorus* spp.]) and the pelagic amphipod (*Macrohectopus branickii*) that would be needed to evaluate this hypothesis (see [52]). The movement to shallower water could be driven by the impact of rising surface water temperatures on the trade-off between growth and reproduction versus predator avoidance. Vertical migration is often viewed as a behavioral adaptation designed to maximize growth and reproduction while minimizing mortality due to predation [14], [51]. Animals that can remain in warmer waters will have a distinct advantage in terms of growth and reproductive rates, but they are also at risk of predation from visually orienting predators during daylight hours [14]. We speculate that increasing temperatures could alter the costs and benefits of staying in shallower water, thus leading zooplankton to occupy shallower depths during daylight hours [15]. Patterns of diel vertical migration are strong for several of the most abundant taxa [48], including *Epischura* [49] and its primary predators *Macrohectopus* [53] and the golomyanka [34]. More work examining the trade-offs for Lake Baikal zooplankton might provide insights into the potential for this mechanism to explain the long-term changes in depth distributions.

Adult copepods were the only zooplankton group for which depth distribution did not change through time. Adult *E. baikalensis* are thought to be able exploit a wide range of phytoplankton, from picoplankton to large diatoms such as *Aulacoseira baicalensis* [54], such that a variety of potential food would have been available across depths. Predation risk, growth and reproduction are weighted differently for adult and juvenile copepods throughout the water column [55], and significant metabolic changes occur across stages [56] that may reasonably affect environmental preferences and tolerances. Preliminary experiments suggest that survivorship of adult *Epischura* decreases at temperatures above 15°C (T. Ozersky unpubl.), while the high abundances of nauplii and copepodites in surface waters during the summer months suggests that these life stages tolerate relatively high temperatures.

### Implications of plankton depth changes in Baikal

Although diatoms shifted to a deeper position in the water column through time it is not clear that they have experienced less beneficial conditions. On average their exposure to light has not changed significantly (Fig. 6), probably because the average depth of diatoms in the 1940 s was relatively deep (∼40 m) where light conditions were already low. In waters deeper than ∼25 m light availability decreases slowly with depth, in comparison with surface waters (Fig 5). Although we do not have nutrient data, we might anticipate that nutrients in the upper stratum are low relative to levels below the thermocline, so it is possible that diatoms experienced slightly better nutrient conditions in the later decades of the time series. The extent to which freshwater diatoms might be mixotrophic is largely unknown, although such flexibility would be favored in conditions of shifting light. If the depth change does not matter much for the diatoms themselves, it may yet matter for the zooplankton that depend on algal resources. Diatoms comprise an important component of the Lake Baikal primary productivity, in terms of both biomass and nutritional content [57], and their dynamics should be relatively important for grazers and the food web more generally [58], [59].

Changes in spatial overlap between grazers and phytoplankton could potentially alter encounter and ingestion frequency by grazers, leading to changes in the composition of the zooplankton diet. As depth distributions changed through time, the spatial overlap of copepods with all phytoplankton increased, while it decreased for cladocerans. However, the mismatch between cladoceran DWA and that for all phytoplankton groups did not appear to negatively affect this group, because their abundance increased significantly through time ([28] and this study). That being said, our overlap analyses have limitations. First, our calculations are based on daytime distributions for zooplankton. Due to vertical migration toward the surface at night, it is likely that nighttime overlap differs significantly from our results. Interestingly, it seems that a move toward shallower waters at night would further segregate phytoplankton and zooplankton, as zooplankton have actually moved to slightly shallower daytime positions than most phytoplankton groups through time. Second, our depth resolution is relatively coarse due to the small number of discrete depth samples collected for phytoplankton (0, 10, 50, 100, and 200 m) and the aggregation of zooplankton across depth strata with a closing net. Given this discrete sampling approach it is possible that we could have missed deep chlorophyll maxima, for example. Third, as previously mentioned, small (<2 µm) autotrophic and heterotrophic plankton were not included in the long-term data, but they are often abundant in summer months. Finally, our analyses are based on data from a single sampling station in Lake Baikal; long-term plankton abundance data from a different Baikal sampling location have similarities that suggest generalities in plankton dynamics in the Southern basin [33], but interpretation of data from a single station should be done with caution.

In summary, this study provides further evidence that the plankton community in Lake Baikal is experiencing significant long-term changes. Our results suggest that the depth distribution of many plankton groups in Lake Baikal has changed dramatically through time. Diatoms now occur deeper, perhaps as a result of sinking due to increased stratification and reduced mixing. Most zooplankton groups, however, shifted to shallower positions in the water column over time. The factors driving the shift of zooplankton to shallower daytime depths are not clear, but we suggest it may be a response to warming surface waters. While we are limited to speculation about mechanisms underlying these changes, we feel it is important to report these patterns, thereby enabling comparisons with other large lakes experiencing surface warming. If similar patterns are found in other systems then perhaps the relevant data (e.g. day and night zooplankton depth distributions) exist to evaluate potential hypotheses. Finally, effects of changes described in this study on higher trophic levels in the Lake Baikal food web are currently unknown because the long-term sampling has focused on plankton. Future studies should examine how changing depth distributions of the plankton might affect energy transfer to organisms at higher trophic levels.
